# Systemic inflammatory burden and local inflammation in periodontitis: What is the link between inflammatory biomarkers in serum and gingival crevicular fluid?

**DOI:** 10.1002/cre2.162

**Published:** 2019-01-24

**Authors:** Alkisti Zekeridou, Andrea Mombelli, Jose Cancela, Delphine Courvoisier, Catherine Giannopoulou

**Affiliations:** ^1^ Division of Periodontology University Clinic of Dental Medicine, University of Geneva Geneva Switzerland

**Keywords:** cytokines, periodontal disease, periodontal treatment, serum

## Abstract

In periodontitis patients, high levels of several inflammatory markers may be expressed in serum, reflecting the effect of local disease on the general health. The objective of the present analysis was to compare cytokine levels assessed in peripheral blood with those in the gingival crevicular fluid (GCF) and evaluate the impact of nonsurgical periodontal therapy on the incidence of high levels of 12 biomarkers in serum. Twenty‐four patients with chronic periodontitis (Group P) contributed with serum and GCF samples at baseline (BL) and 1 and 3 months after periodontal treatment (M1 and M3). Samples were assessed for 12 cytokines using the Bio‐Plex bead array multianalyte detection system. For each analyte, peak values were calculated as greater than the mean + 2*SD* of the one found in 60 periodontally healthy participants. Significant correlations between serum and GCF values were obtained in the periodontitis group for interleukin (IL) 1ra, IL‐6, and interferon γ at BL and for macrophage inflammatory protein 1β at M3 after treatment. Periodontitis subjects were found to exhibit high peaks for several inflammatory markers in serum. The highest incidence of peaks at BL was found for interferon γ (37.5% of the periodontitis subjects). For the four biomarkers with a detection frequency of >75% at BL (IL‐1ra, IL‐8, macrophage inflammatory protein 1β, and vascular endothelial growth factor), no significant difference was observed over time for the P group or between the two groups at BL. The significant correlation found between the serum and the GCF for certain cytokines and the fact that periodontitis subjects exhibit high peaks for several inflammatory markers in serum may support the hypothesis that the inflammatory reaction due to periodontitis is not restricted to the diseased sites. Within the limitations of the study, periodontal therapy did not seem to have any significant impact on the systemic cytokine levels.

## 1 INTRODUCTION

Periodontitis is an inflammatory disease that affects the supporting tissues of the tooth. Bacteria contamination activates the immune response of the host (Kinane, 2001), and both bacterial products and inflammatory mediators are released into the bloodstream, resulting in a low‐grade inflammatory state (Buduneli & Kinane, 2011; Loos & Tjoa, 2005). Chronic inflammation has been proposed as one of the main mechanisms linking periodontitis with a number of systemic conditions such as cardiovascular disease (Dave & Van Dyke, 2008), preterm low‐weight birth babies (Offenbacher et al., 1996), diabetes (Chapple et al., 2013), and respiratory problems (Hayes, Sparrow, Cohen, Vokonas, & Garcia, 1998).

As a first step to the comprehension of the link of periodontal to general health, research in serum levels of different inflammatory biomarkers was conducted by different groups. There are numerous studies showing that markers of inflammatory response are elevated in subjects suffering from periodontitis compared with healthy ones (Bretz et al., 2005; Passoja et al., 2010; Robati, Ranjbari, Ghafourian Boroujerdnia, & Chinipardaz, 2011). In the same direction, in order to understand the effect of periodontitis in various systemic conditions, studies were conducted in specific groups presenting comorbidities (Perunovic, Rakic, Nikolic, et al., 2016; Satpathy et al., 2015; Zhu, Lin, Zheng, & Chen, 2015).

Many studies evaluated simultaneously different biomarkers in the gingival crevicular fluid (GCF) and in the bloodstream, based on the hypothesis that chronic periodontal inflammation induces a chronic systemic inflammatory status. In a recent study, in a cohort of 60 patients divided in three equal groups (healthy, gingivitis, and periodontitis), the concentration of GCF and serum interleukin (IL) 35 was found significantly higher in the periodontitis group (Raj et al., 2018). The same results were found for other inflammatory markers, such as visfatin, vascular endothelial growth factor (VEGF), oncostatin M, protein carbonyl, and tumor necrosis factor α (TNF‐α). All of them were found higher in the periodontitis groups compared with healthy ones, in both serum and GCF; visfatin, VEGF, oncostatin M, and TNF‐α decreased, in both fluids, after scaling and root planning (Baltacioglu, Akalin, Alver, Deger, & Karabulut, 2008; Gokul, Faizuddin, & Pradeep, 2012; Pradeep et al., 2011; Pradeep, Prapulla, Sharma, & Sujatha, 2011; Raghavendra et al., 2012; Thorat, Pradeep, & Garg, 2010; Turer, Durmus, Balli, & Guven, 2017). In another study that investigated patients with ischemic stroke, the authors found higher concentrations of some cytokines in the serum in the ischemic patients' group but no correlation between the GCF concentrations and the serum ones (Wytrykowska, Prosba‐Mackiewicz, & Nyka, 2016). The same results, with no association in serum and GCF, though there were higher concentrations in GCF of periodontitis patients, were also reported for different cytokines in a cohort of pregnant women (Fiorini et al., 2012). Poor link between the levels of serum and GCF markers was also found in a pilot study in obese people (Fell, Zee, & Arora, 2013).

A number of intervention studies also tried to contribute with additional insight on the link between periodontal infections and systemic health. More precisely, they examined whether periodontal treatment contributes to favorable changes of the levels of systemic inflammatory markers. Different protocols, in terms of studying the short‐term or long‐term response to periodontal treatment, and a variety of systemic markers, such as acute‐phase proteins, inflammatory cytokines, or measures of endothelial dysfunction, were assessed (Chen et al., 2012; D'Aiuto, Nibali, Parkar, Suvan, & Tonetti, 2005; D'Aiuto, Parkar, Andreou, et al., 2004; Morozumi et al., 2018; Turer et al., 2017; Zhou, Duan, Hu, & Ouyang, 2013). A recent systematic review and meta‐analysis conducted by Teeuw et al. (2014) reported that periodontal treatment improves endothelial function and reduces inflammatory markers, especially in those individuals suffering from both periodontitis and cardiovascular disease or diabetes mellitus. However, in a large group of pregnant women, periodontal treatment had no influence on the levels of the selected inflammatory biomarkers (Michalowicz et al., 2009). These findings were further confirmed by many others who did not detect differences in systemic biomarkers before and after treatment (Fiorini et al., 2013; Geisinger et al., 2016). Another systematic review and meta‐analysis on the role of C‐reactive protein rejected the hypothesis that periodontal treatment can reduce systemic C‐reactive protein levels (Ioannidou, Malekzadeh, & Dongari‐Bagtzoglou, 2006). Several factors, such as treatment protocol, patient characteristics, severity of periodontitis, and presence of other comorbidities, may be responsible for the observed heterogeneity between the studies.

In the literature, changes of serological markers after periodontal therapy have been reported mainly as means or medians. This way of looking at the data may however mask relevant pathological effects that concern only a few individuals. Our group has shown that patients with untreated periodontitis in fact sometimes display very high serum levels of multiple inflammatory biomarkers simultaneously (Almaghlouth et al., 2011). In a follow‐up investigation, we found that nonsurgical periodontal treatment had the potential to reduce most of these peak levels (Almaghlouth et al., 2014).

Measuring the levels of 12 cytokines, as sample biomarkers, in the GCF of patients with periodontitis at diseased and healthy teeth and GCF levels in healthy individuals, we found significant differences between periodontally diseased or healthy persons rather than diseased or healthy sites (Zekeridou, Giannopoulou, Cancela, Courvoisier, & Mombelli, 2017), suggesting a generalized inflammatory state that is not limited to clinically diseased sites. To further investigate possible associations between the systemic inflammatory burden and local inflammation in periodontitis, the objective of the present analysis was to compare cytokine levels assessed in peripheral blood with those in the GCF. In addition, we evaluated the impact of nonsurgical periodontal therapy on the incidence of high levels of 12 biomarkers in serum.

## 2 MATERIAL AND METHODS

The study was approved by the Ethical Committee of the University Hospitals of Geneva, Geneva, Switzerland, and conducted according to the principles outlined in the Declaration of Helsinki on human medical experimentation (number of reference 13‐185). Informed written consent form was obtained from each participant.

From October 2013 to March 2016, we recruited 84 participants among persons seeking dental treatment at the University Dental Clinic of the University of Geneva. Participants had to be in good general health (no history of systemic diseases) and aged between 30 and 70 years. Twenty‐four individuals were diagnosed with chronic periodontitis (Armitage, 1999), based on the presence of at least two sites with probing pocket depth (PD) ≥5 mm, clinical attachment loss ≥1 mm, and radiographic evidence of marginal bone loss, and were assigned to Group P. Sixty periodontally healthy individuals, with no sites with PD >4 mm, or clinical attachment loss ≥1 mm, or radiographic evidence of bone loss, were assigned to Group H. Periodontal PD was assessed in millimeters with a periodontal probe (PCP12, Hu‐Friedy, Frankfurt am Main, Germany) with applying light pressure into the gingival crevice.

The clinical protocol of this study has been presented in detail in a previous paper focusing on site specificity of GCF assessments (Zekeridou et al., 2017). The participants in Group P received nonsurgical periodontal treatment according to the standard operating procedures of the institution (Mombelli, Jr, Walter, & Wetzel, 2015). All diseased sites were treated with deep scaling and root planning under local anesthesia. The treatment was delivered in one to four sessions depending on the extent of the disease. During therapy plus seven additional days, these patients rinsed their mouth twice a day with 0.2% chlorhexidine. No other medication was prescribed. The patients were again seen after 1 (M1) and 3 months (M3). The participants of Group H received no periodontal therapy and were examined only once.

At baseline (BL) in both groups and during the two follow‐up visits in Group P, 5 ml of blood was drawn by venipuncture using tubes without anticoagulant. The blood specimens were immediately centrifuged for 10 min at 1,300 *g*. Two milliliters of serum were stored at −20°C until the day of the analysis. In addition, samples of GCF were obtained with Durapore® (Millipore, Bedford, MA, USA) membrane with a pore size of 0.22 μm. Strips of 2 × 6 mm were placed at the gingival crevice as previously reported (Zekeridou et al., 2017). The specimens were stored in microtubes at −20°C until further processing. In Group P, two periodontally diseased (PP) sites (PD ≥5 mm) and two periodontally healthy (PH) sites (PD ≤3 mm) were sampled individually. In Group H, the samples were obtained from two healthy sites (HH) with PD ≤3 mm.

The plaque index and gingival index (Loe, 1967), PD, bleeding on probing, and recession were recorded at six sites per tooth of every tooth at BL for the two groups and in addition at M1 and M3 for the periodontitis group.

### 2.1 Analysis of inflammatory biomarkers

Twelve biomarkers were assessed in serum and GCF using a multiplex fluorescent bead‐based immunoassay and the Bio‐Plex 200 Suspension Array System (Bio‐Rad Laboratories, Hercules, CA, USA). The panel (kit L5000HIVS1, Bio‐Rad Laboratories) included IL‐1β, IL‐1 receptor antagonist (IL‐1ra), IL‐6, IL‐7, IL‐8, IL‐17, basic fibroblast growth factor (b‐FGF), granulocyte colony‐stimulating factor (G‐CSF), granulocyte macrophage colony‐stimulating factor (GM‐CSF), interferon γ (IFN‐γ), macrophage inflammatory protein 1β (MIP‐1β), TNF‐α, and VEGF. The assays were performed according to the manufacturer's instructions. The limit of detection was 1 pg/ml.

### 2.2 Statistical analysis

Patient averages were computed for all variables assessed at multiple sites per participant. Longitudinal changes in Group P were analyzed with the Wilcoxon matched‐pairs signed‐rank test for the serum and the GCF samples. Differences between Groups P and H at BL were analyzed with Fisher exact test for categorical variables and Wilcoxon rank sum test for continuous variables. Correlations between serum and the GCF levels were evaluated by Spearman correlations.

To assess the simultaneous presence of multiple inflammatory biomarkers at a very high level in the serum of Group P, we dichotomized the value of each reading of each biomarker into normal or extreme. The cutoffs were defined as greater than the mean plus 2 standard deviations (mean + 2*SD*) found in the analyses of serum in Group H. Two of these 12 cytokines (G‐CSF and GM‐CSF) were not detected in any serum sample of these 60 periodontally healthy persons. Five other of these 12 biomarkers had less than 25% of patients with detectable values. For these seven cytokines, each positive reading was considered a peak (i.e., the cutoff was set at the respective level of detection).

The number of peaks at each time point for Group P was compared using Fisher exact test. McNemar's exact test was applied to 2 × 2 contingency tables tabulating the number of peaks at BL and M1 after treatment and BL and M3 after treatment for each individual. In addition, for the analytes with a detection frequency of >75%, we calculated the mean concentration and its standard deviation.

The statistical software R (version 3.4.1; The R Foundation for Statistical Computing, Vienna, Austria) was used for the analyses. *P* values of <0.05 were accepted for statistical significance.

## 3 RESULTS

The demographics and BL clinical parameters of the 24 patients of Group P and the 60 healthy controls are shown in Table 1. All parameters were significantly higher in Group P than Group H (*P* < 0.05). Sixteen of 24 patients (66%) in the P group were diagnosed with severe chronic periodontitis on the basis of the presence of PD ≥7 mm at multiple teeth.

**Table 1:** Demographic data and full‐mouth clinical parameters at baseline

	Periodontitis patient	Healthy participant	
Clinical parameter	(*n* = 24)	(*n* = 60)	*P* value
Sex			
M	14 (58.3)	32 (53.3)	0.081
F	10 (41.7)	28 (46.7)	
Smoking			
Yes	15 (62.5)	59 (98.3)	**<0.001**
No	9 (37.5)	1 (1.7)	
Age (years)	51.6 ± 10.3	36.9 ± 12.2	**<0.001**
	(*n* = 24)	(*n* = 40)	
% sites with PI > 0	77.1 ± 27.5	32.5 ± 33.5	**0.003**
% sites with GI > 0	71.9 ± 25.9	12.5 ± 22.2	**<0.001**
% sites with BOP	70.8 ± 26.2	10.0 ± 20.5	**<0.001**
Mean PD (mm)	5.1 ± 0.9	2.3 ± 0.5	**<0.001**
Mean REC (mm)	0.8 ± 0.7	0.3 ± 0.6	**<0.001**

*Note*. Bold values represent statistically significant difference between two groups. Data are the number of participants (percentage) or mean ± standard deviation. PI: plaque index; GI: gingival index; BOP: bleeding on probing; REC: recession.

Three serum specimens were available from all 24 participants of Group P (BL, M1, and M3). Thus, the levels of 12 biomarkers (cytokines) were assessed in 72 specimens from the diseased patients. In addition, the same cytokines were assessed in one serum sample of each of the 60 participants of Group H.

**Figure 1:**
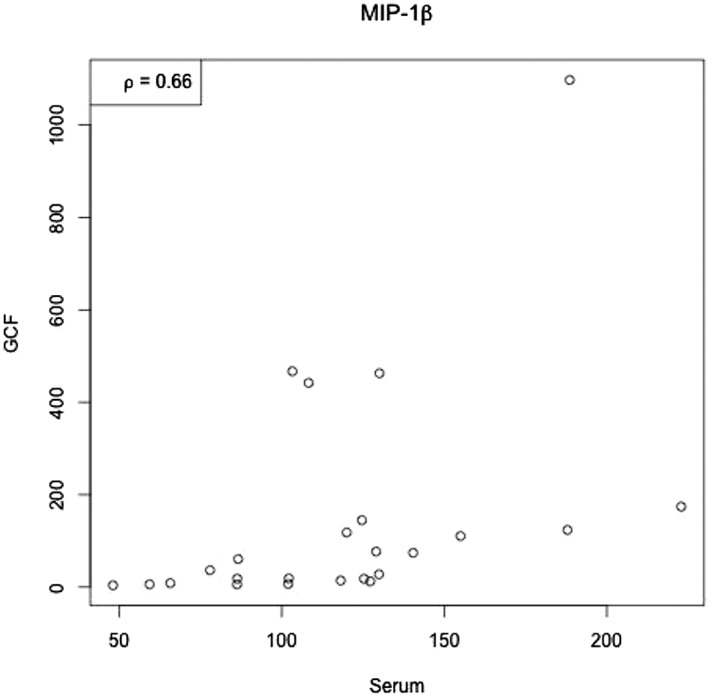
Range of the values in pg/ml and correlation between serum and gingival crevicular fluid (GCF) for macrophage inflammatory protein 1β (MIP‐1β) at 3 months

Correlations between serum and GCF values in the healthy and periodontitis participants at the different time points are presented in Table 2. Correlations were significant in the periodontitis group for IL‐1ra, IL‐6, and IFN‐γ at BL and for MIP‐1β at M3. Figure 1 shows the range of the values and the correlation between serum and GCF for MIP‐1β at 3 months.

**Table 2:** Correlation between serum and gingival crevicular fluid cytokine values in healthy and periodontitis participants at baseline

	Group H (*N* = 20)		Group P (*N* = 24)					
Biomarker (pg/ml)	Baseline		Baseline		1 month (M1)		3 months (M3)	
	Correlation	*P*	Correlation	*P*	Correlation	*P*	Correlation	*P*
IL‐1β	−0.30	0.20	NA	NA	NA	NA	0.08	0.71
IL‐1ra	0.42	0.07	0.49	**0.02**	0.19	0.38	−0.16	0.46
IL‐6	−0.27	0.25	−0.51	**0.01**	0.11	0.63	0.18	0.39
IL‐8	0.13	0.58	−0.20	0.36	−0.12	0.58	0.00	1.00
IL‐17	−0.33	0.15	0.02	0.94	0.01	0.96	0.23	0.29
b‐FGF	0.06	0.80	NA	NA	0.08	0.71	0.24	0.26
G‐CSF	NA	NA	NA	NA	NA	NA	NA	NA
GM‐CSF	NA	NA	NA	NA	NA	NA	NA	NA
IFN‐γ	0.22	0.35	0.54	**0.01**	0.23	0.29	−0.33	0.11
MIP‐1β	0.02	0.92	−0.28	0.18	−0.29	0.18	0.66	**0.001**
TNF‐α	−0.22	0.35	0.17	0.42	0.19	0.38	0.25	0.24
VEGF	0.35	0.13	0.09	0.66	0.17	0.44	0.01	0.95

*Note*. Bold values represent statistically significant correlation. NA: Correlation could not be estimated because serum values were all below detection threshold. b‐FGF: basic fibroblast growth factor; G‐CSF: granulocyte colony‐stimulating factor; GM‐CSF: granulocyte macrophage colony‐stimulating factor; IFN: interferon; IL: interleukin; MIP: macrophage inflammatory protein; TNF: tumor necrosis factor; VEGF: vascular endothelial growth factor.

Table 3 shows the number of positive samples of the 12 biomarkers in the 60 periodontally healthy subjects and, if detected in at least 25% of the samples, the mean values, standard deviations, and mean ± 2*SD*, which served as a threshold to define the peak values in the periodontitis patients. Two cytokines could not be detected in any serum sample (G‐CSF and GM‐CSF) and five molecules were detected in a frequency of less than 25% (IL‐1β, IL‐6, IL‐17, b‐FGF, and IFN‐γ). For the remaining five biomarkers, the cutoff value was calculated (mean ± 2*SD*) in order to define the peak values for each patient.

**Table 3:** Detection frequency of 12 analytes in 60 periodontally healthy persons, means in pg/ml, and, if detected in more than 15 (25%) cases, standard deviation (SD) and mean + 2*SD*

Biomarker (pg/ml)	*N* (%)	Mean (*SD*)	Mean + 2*SD* (cutoff)
IL‐1β	2 (3.3%)	—	0.1^a^
IL‐1ra	55 (91.7%)	17.7 (25.9)	69.6
IL‐6	7 (11.7%)	—	0.1^a^
IL‐8	46 (76.7%)	8.5 (9.5)	27.5
IL‐17	5 (8.3%)	—	0.1^a^
b‐FGF	1 (1.7%)	—	0.1^a^
G‐CSF	0 (0%)	—	0.1^a^
GM‐CSF	0 (0%)	—	0.1^a^
IFN‐γ	6 (10.0%)	—	0.1^a^
MIP‐1β	60 (100%)	129.5 (59.7)	248.9
TNF‐α	24 (40.0%)	10.3 (25.6)	61.5
VEGF	56 (93.3%)	192.5 (161.5)	515.5

*Note*. Mean + 2*SD* values served as a threshold to define peak values in periodontitis patients. b‐FGF: basic fibroblast growth factor; G‐CSF: granulocyte colony‐stimulating factor; GM‐CSF: granulocyte macrophage colony‐stimulating factor; IFN: interferon; IL: interleukin; MIP: macrophage inflammatory protein; TNF: tumor necrosis factor; VEGF: vascular endothelial growth factor.

a For these biomarkers with <25% of samples with detectable values, the threshold was set at the detectable level (0.1).

Table 4 shows the incidence (number and frequency) and the range of serum peak values of the 12 cytokines in periodontitis patients before and 1 and 3 months after periodontal therapy. For example, at BL, four patients presented peak values in the serum for IL‐1ra, three for IL‐6, and nine for IFN‐γ. At M1, still, three patients presented peak values for IL‐1ra, six for IL‐6, and five for IFN‐γ, whereas at M3, the respective values were six, three, and three subjects for those cytokines. The highest incidence of peaks at BL was found for IFN‐γ (37.5% of the periodontitis subjects). For this specific biomarker, these peaks decreased slightly at M1 (20.8%) and decreased even further and significantly at M3 (12.5% of the patients). Summing the serum peaks across biomarkers and patients, at BL, 19 peak values were observed. These numbers remained approximately stable over time (21 peaks at M1 and 22 peaks at M3 after treatment).

**Table 4:** Incidence (number and frequency) and range of peak values in pg/ml (readings above mean + 2*SD* of healthy persons) of 12 cytokines in periodontitis patients before and 1 and 3 months after therapy

Biomarker (pg/ml)	Baseline		1 month		3 months	
	*N* (%)	Range	*N* (%)	Range	*N* (%)	Range
IL‐1β	0	—	0	—	3 (12.5%)	0.9–5.5
IL‐1ra	4 (16.7%)	71.4–149.2	3 (12.5%)	77.1–141.3	6 (25.0%)	80.1–140.3
IL‐6	3 (12.5%)	0.2–1.8	6 (25.0%)	02–2.2	3 (12.5%)	5.6–8.3
IL‐8	0	—	0	—	1 (4.2%)	29.3
IL‐17	1 (4.2%)	6.0	3 (12.5%)	28.0–55.9	1 (4.2%)	23.0
b‐FGF	0	—	2 (8.3%)	14.4–28.7	2 (8.3%)	4.7–8.0
G‐CSF	0	—	0	—	0	—
GM‐CSF	0	—	0	—	0	—
IFN‐γ	9 (37.5%)	0.7–24.7	5 (20.8%)	0.7–27.6	**3 (12.5%)**	4.2–33.5
MIP‐1β	0	—	0	—	0	—
TNF‐α	0	—	0	—	0	—
VEGF	2 (8.3%)	594.9–710.2	2 (8.3%)	640.2–683.2	3 (12.5%)	671.0–1437.9
Total	19		21		22	

*Note*. Values in bold and underlined significantly changed compared to baseline. b‐FGF: basic fibroblast growth factor; G‐CSF: granulocyte colony‐stimulating factor; GM‐CSF: granulocyte macrophage colony‐stimulating factor; IFN: interferon; IL: interleukin; MIP: macrophage inflammatory protein; TNF: tumor necrosis factor; VEGF: vascular endothelial growth factor.

**Figure 2:**
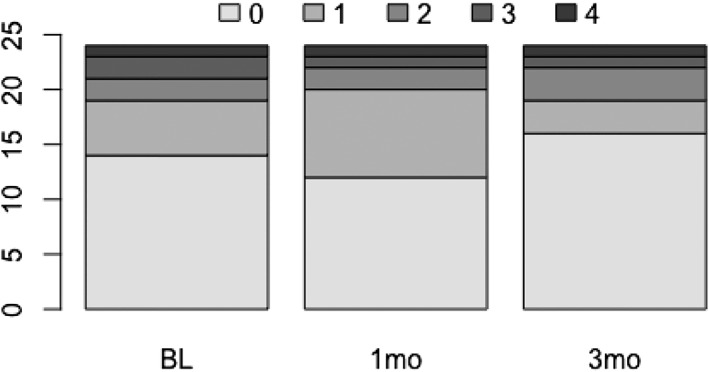
Number of biomarker peaks per person at baseline (BL), 1 month, and 3 months after therapy

As shown in Figure 2, at the patient's level, there was no significant association of having at least one peak at BL with having at least one peak either at M1 (*P* = 0.77) or at M3 (*P* = 0.68).

Focusing on the levels of four biomarkers with a detection frequency of >75% at BL (IL‐1ra, IL‐8, MIP‐1β, and VEGF), there was no significant difference over time in the periodontitis group or between healthy and periodontitis subjects at BL.

## 4 DISCUSSION

The present study evaluated the effect of initial periodontal therapy on the expression of 12 selected biomarkers in the serum of patients with chronic periodontitis. A group of 60 periodontally healthy subjects served as control. Correlations with the previously reported levels in the GCF of the same patients were further assessed (Zekeridou et al., 2017).

Periodontitis is an inflammatory disease with increasing evidence suggesting that the disease itself and its treatment are associated with several impaired health conditions, such as cardiovascular disease, diabetes, adverse pregnancy outcomes, and respiratory diseases (Teeuw et al., 2014; Teeuw, Gerdes, & Loos, 2010). The expression of the 12 selected biomarkers was assessed in the serum of a group of periodontitis patients before and 1 and 3 months after nonsurgical periodontal treatment and was compared with that in periodontally healthy persons. We used the Bio‐Plex 200 Suspension Array System, a technique that permits to simultaneously identify and quantify up to 100 different molecules even when very small volumes are available, such as the GCF. The high sensitivity and accuracy offered by this technique have helped to broaden the understanding of the complex immune reaction (Elshal & McCoy, 2006; Leng et al., 2008) in periodontitis and other systemic diseases.

Driven by the increasing evidence that periodontal disease has a general effect on the health status of the patients, we aimed to identify biomarkers that are correlated on the local diseased sites and the peripheral blood. In the present analysis, three biomarkers, IL‐1ra, IL‐6, and IFN‐γ, were significantly correlated in serum and GCF at BL and one biomarker, the MIP‐1β, at 3 months. In a study investigating the expression of cytokines in GCF and serum of people with inflammatory bowel disease and periodontitis, the authors found strong correlation for two of the analytes in serum and GCF (Figueredo et al., 2011). These correlations suggest an effect of the local diseased sites to the general status and via versa. Other studies also found correlations between the local and the general markers verifying our suggestion for the link between them (Noack et al., 2017; Zheng, Chen, Shi, Jepsen, & Eberhard, 2006). There is still, though, great variety in the results in the literature with other studies failing to prove association between GCF and blood biomarkers (Fell et al., 2013; Fiorini et al., 2012; Wytrykowska et al., 2016). Different methodologies, characteristics of the groups, and various selection criteria can justify this great diversity.

Furthermore, on the basis of our previous studies showing that periodontitis patients present peak values for several cytokines in the serum simultaneously, we focused our analysis on peaks rather than mean or median values (Almaghlouth et al., 2014; Giannopoulou et al., 2016). The majority of periodontitis patients presented no peaks at BL (14 out of the 24), half of the remaining 10 subjects presented one peak, two subjects presented two peaks, and two others presented three peaks simultaneously. Only one subject presented four peaks. No significant change was observed neither at M1 nor at M3 after nonsurgical periodontal treatment, as the sum of peaks remained approximately the same after treatment (BL = 19, M1 = 21, and M3 = 22). In our previous study, we reported that the majority of subjects (66 out of 80) presented at least one peak in the serum at BL and that periodontal therapy greatly reduced the number of patients with peaks, especially those presenting three or more peaks before treatment. The results remained stable 12 months after the initial therapy. Antibiotics and further surgical treatment did not enhance this effect. Comparisons with the present findings may be difficult, as periodontitis was less severe and the panel of acute‐phase proteins was not included in the present study.

The incidence of serum peaks was not similar for all the biomarkers. Among the 12 cytokines, seven had undetectable concentrations at BL (IL‐1β, IL‐8, b‐FGF, G‐CSF, GM‐CSF, MIP‐1β, and TNF‐α), a finding that suggests that these specific biomarkers are not the most appropriate when studying the relationship of periodontal disease and/or periodontal treatment with the systemic response. However, sporadically, after periodontal treatment, some peaks appeared in the serum of some patients, such as IL‐1β at M3 in three patients, b‐FGF at M1 and M3 in two patients, and IL‐8 at M3 in one patient. Thus, we detected only five biomarkers with peak values in the periodontitis group: IL‐1ra, IL‐6, IL‐17, IFN‐γ, and VEGF. These biomarkers also presented serum peak values in our previous study using a similar protocol to the present one (Almaghlouth et al., 2014). Only one biomarker, IFN‐γ, was frequently found at very high levels (*n* = 9 at BL), and its incidence gradually decreased at M1 (*n* = 5) and at M3 (*n* = 3) after treatment. In general, no patient presented an unusual pattern of peaks, which could be in part explained by the fact that in the inclusion criteria, any comorbidities that could affect the immune system were excluded. However, the presence of undetected medical condition that could contribute in some peak inflammatory values cannot be ruled out.

As previously mentioned, we found variation in the number of peaks after periodontal treatment with some of them increasing immediately after at 1 month and decreasing again at 3 months and via versa. When analyzing a large number of inflammatory markers in response to periodontal treatment, great heterogeneity has been reported (Behle et al., 2009). Our results corroborate with these findings and strengthen the multifactorial concept of periodontal disease and its treatment. Large variations and heterogeneous responses to periodontal therapy have been reported when one or two specific biomarkers are assessed. For example, some studies showed significant increase in the levels of TNF‐α and IL‐6 (D'Aiuto et al., 2005; Ide et al., 2004) immediately after therapy, whereas others found that treatment had no effect on the serum levels of the same analytes (Yamazaki et al., 2005). It should be kept in mind that among different studies, the clinical protocols used are not similar, the study populations are different, the extent and severity of periodontitis differ, and factors such as the presence of specific pathogens are not always taken in consideration. However, all these studies, together with ours, emphasize the preexisting susceptibility for systemic inflammation and response to treatment.

The main limitation of our study is that the clinical evaluation performed after 3 months included only the presence of plaque and inflammation indices; thus, the correlations between the cytokine levels of serum and GCF and the clinical parameters, mainly the pocket probing depth (PD), could not be assessed. Because of the lack of these clinical data, the success of the treatment cannot be completely evaluated. One can argue that the absence of an effect of the treatment on the serum level of the 12 examined cytokines might also be explained by a probable nonsuccessful periodontal therapy. The treatment was performed following the standard protocol of initial periodontal therapy of our division, aiming to a positive therapeutic result, but the above assumption cannot be ruled out. Our previous findings showed that most of the selected biomarkers could discriminate between healthy and diseased individuals but not between healthy and diseased sites in the same individual. Although no causality can be proven, these results, being reinforced by the correlations between local and systemic biomarkers, suggest that there is a general effect of periodontal disease on systemic health that is not limited in the mouth.

In conclusion, by analyzing local and systemic levels of various cytokines, we reinforce the hypothesis that periodontitis is an inflammatory disease that awakes the immune response with a cascade of inflammatory markers that are not limited in the diseased sites. In our study, periodontitis patients presented high peaks for a limited number of the selected inflammatory markers in serum, but periodontal treatment had only minor effect on these peaks. Future research should focus on identifying the biomarkers that best reflect those interactions and explore in which level periodontal treatment can ameliorate the local and the general inflammatory status of the patient.

## CONFICT OF INTEREST

None declared.

## AUTHOR CONTRIBUTION

CG and AM contributed to conception, design, data acquisition, analysis, and interpretation and drafted and critically revised the manuscript. AZ managed the recruitment of the subjects, performed periodontal treatment and data collection, and drafted and critically revised the manuscript. JC performed sample collection and analysis and critically revised the manuscript. DC contributed to data analysis and critically revised the manuscript.
